# Prevalence of knowledge, attitudes, and practices regarding antimicrobial resistance in Africa: a systematic review and meta-analysis

**DOI:** 10.3389/fmicb.2024.1345145

**Published:** 2024-03-22

**Authors:** Yibeltal Assefa Atalay, Kelemu Abebe Gelaw

**Affiliations:** ^1^School of Public Health, College of Health Science and Medicine, Wolaita Sodo University, Wolaita Sodo, Ethiopia; ^2^School of Midwifery, College of Health Science and Medicine, Wolaita Sodo University, Wolaita Sodo, Ethiopia

**Keywords:** prevalence, knowledge, attitude, practice, antimicrobial resistance, systematic review, meta-analysis, Africa

## Abstract

**Background:**

One of the main threats to public health today is antibiotic resistance. This resistance leads to the persistence of infections in the body. It poses an increased risk of transmission to humans and animals through various routes, such as food, water, and the environment.

**Objectives:**

This study aimed to ascertain the overall prevalence of knowledge, attitudes, and practices regarding antimicrobial resistance in Africa.

**Methods:**

A systematic review and meta-analysis of published and unpublished studies was conducted in Africa according to the Preferred Reporting Items for Systematic Reviews and Meta-Analyses (PRISMA) guidelines. Searches were conducted using appropriate search terms in PubMed, Web of Science, Science Direct, Google Scholar, African Journals Online, and the Cochrane Library. Data were extracted using Microsoft Excel, and STATA version 14 was used for analysis. Publication bias was checked by funnel plot, Egger, and Begg regression tests. A *p*-value of 0.05 was regarded to indicate potential publication bias. Using I2 statistics, the heterogeneity of the studies was evaluated. Using forest plots, the random effect model was used to present the pooled prevalence with a 95% confidence interval (CI) of meta-analysis.

**Results:**

This review included 39 studies, with 18,769 study participants. Among these 39 studies, 38 were on knowledge assessment, 28 on attitude assessment, and 25 on good practice assessment towards antimicrobial resistances. The overall pooled prevalence level of knowledge regarding antimicrobial resistance in Africa was 55.33% (95% CI: 47.48, 63.18). The overall pooled prevalence of positive attitudes toward antimicrobial resistance in Africa was 46.93% (95% CI: 35.10, 58.76), and the overall pooled prevalence of good practice of antimicrobial resistance in Africa was 51.05% (95% CI: 45.24, 56.87). In addition, sub-group statistical analysis was performed in this meta-analysis, stratified by population sub-region and study design types.

**Conclusion:**

In Africa, the pooled prevalence of knowledge, attitudes, and practices regarding antimicrobial drug resistance among different groups, including the general population, patients, tertiary school students, healthcare workers, and animal owners was found to be low level. Therefore, it is imperative to enhance the education and training programs regarding antibiotic resistance for various groups including the general public, patients, students, healthcare workers, and individuals responsible for the well-being of animals.

## 1 Introduction

Antibiotics are a class of medications specifically designed to fight bacterial infections. When used correctly, they can be highly effective in treating various bacterial diseases and, in some cases, even be life-saving (Africa CD, 2018). Antibiotic resistance has emerged as one of the most significant public health challenges. This resistance leads to the persistence of infections in the body and poses an increased risk of transmission to humans and animals through various (Bennani et al., 2020).

The overuse of antimicrobials worldwide has exacerbated the problem of antimicrobial resistance (Llor and Bjerrum, 2014). The emergence of antimicrobial resistance is mainly attributed to the misuse and overuse of antimicrobials in healthcare and agriculture. In addition, antimicrobial resistance can also arise through spontaneous evolution, pathogen mutations, and the transfer of resistant genes through horizontal gene transfer (Dadgostar, 2019). According to the Food and Agriculture Organization’s antimicrobial drug resistance action plans for Rome, Italy (2016–2020), the consequences of antimicrobial resistance are felt in all sectors, regardless of their economic status or geographical location (FAO, 2016).

The global rise in antimicrobial resistance is related to higher mortality rates, besides the spread of resistant strains within and between healthcare facilities. This has also led to greater demand for limited patient care resources. Despite efforts to raise awareness and implement antimicrobial management policies, the prevalence of resistant strains causing community-associated infections continues to increase, while developing new antimicrobials remains limited (Moody et al., 2012).

Furthermore, Antimicrobial resistances pose a severe threat to the well-being of the general public and have resulted in adverse outcomes like serious illness, prolonged hospital stays, persistent disability, increasing healthcare expenditure, an overburdened public healthcare system, increased costs of alternative medications and treatments, ineffectiveness, and increased mortality rates (World Health Organization, 2019). It is also a significant problem for many low and middle-income African countries, associated with poverty, considerable incidence of infectious diseases, and unregulated use of antimicrobials in animals and humans (Fasina et al., 2020).

According to the United Nations Sustainable Development Cooperation Framework guidelines and the problem of antibiotic resistance, the escalation of AMR levels will hinder progress on several sustainable development goals. This challenge is especially noticeable in the objectives that prioritize the enhancement of health and well-being, the alleviation of poverty, the assurance of food security, the promotion of environmental well-being, and the fostering of socio-economic development (Ren and Feng, 2021).

In 2016, the UK government launched an AMR assessment, which found that bacterial infections that cannot be effectively treated due to antibiotic resistance are responsible for a mortality rate of as many as 700,000 people per year worldwide. It is expected that these infections will kill 10 million people every year by 2050 (O'Neill, 2014). Moreover, the World Bank has predicted that failure to address antimicrobial drug resistance could result in the world economy’s annual gross domestic product decline by approximately 4% by 2050. These impacts will likely be more severe in developing countries (Jonas et al., 2017).

A greater prevalence of antimicrobial resistance has been documented in countries with inadequate knowledge, attitudes, and practices toward resistance (Grigoryan et al., 2007). While healthcare providers bear significant responsibility in combating antibiotic resistance due to their ability to prescribe antibiotics and promote patient adherence, it is essential to recognize the critical role that patients, ranchers, and healthcare workers play in the fight against antibiotic resistance. These individuals are also crucial players in the fight against antibiotic resistance as they can take action to prevent the misuse of antibiotics in their respective areas. By working together, healthcare providers, patients, pet owners, and healthcare workers can effectively address the growing threat of antibiotic resistance (Afzal Khan et al., 2013).

As far as we know, there have been few comprehensive systematic reviews and meta-analyses on antibiotic resistance in Africa. Therefore, we aimed to bring together the current data on good knowledge, positive attitudes, and good practices related to antimicrobial resistance. Identifying gaps and improving the understanding and practices of the public, patients, animal owners, and healthcare professionals is crucial to addressing the ongoing rise in antimicrobial resistance. Consequently, the primary outcomes of this review might help policymakers, researchers, and stakeholders, encouraging them to strengthen collaborative efforts across sectors to optimize knowledge, attitudes, and practices related to antimicrobial resistance. In addition, this study will serve as a basis for developing national and international strategies, protocols, and guidelines for preventing and controlling antimicrobial resistance in different settings.

## 2 Methods

### 2.1 Study protocol registration and reporting

When conducting the systematic review, we followed the guidelines of the Preferred Reporting Items for Systematic Reviews and Meta-Analyses (PRISMA) criteria (Liberati et al., 2009; Supplementary Table S1).

### 2.2 Searching strategy

The search was conducted in various databases, including PubMed, Web of Science, Science Direct, Google Scholar, African Journals Online, and the Cochrane Library. The scope of the literature search was limited to English-language studies examining knowledge, attitudes, and practices related to antimicrobial resistance in Africa. A systematic approach was followed to conduct an advanced search on PubMed. Initially, search terms were formulated for four key areas: “antimicrobial resistance,” “knowledge,” “attitudes,” and “practices.” These keywords were retrieved from Google Scholar and then individually searched in PubMed to identify relevant MeSH terms within the MeSH hierarchy tree. Searching strategies were established by using Boolean operators (“OR” or “AND”) to bring the concepts together effectively. The search was also done by combining the above search terms with the names of all countries included in Africa. The search period was from July 1/2023 to August 10/2023.

### 2.3 Condition, context, and population frameworks

Condition (Co): This comprehensive review and meta-analysis included studies that assessed knowledge, attitudes, and practices related to antimicrobial resistance. Context/settings (Co): All observational epidemiological studies conducted in Africa. Population/participants (P): patients, the general population, healthcare workers, pet owners, and students.

### 2.4 Inclusion and exclusion criteria

This study included various research articles, including full-text, English-language, published and unpublished, cross-sectional, case–control, and cohort studies. Excluded from the analysis were duplicate sources, intervention studies, case reports, systematic reviews, qualitative articles, case series, conference abstracts, letters to the editor, and any article that remained inaccessible after at least two email exchanges with the primary author. The COCOPOP (Condition, Context, and Population) paradigm was utilized to determine the suitability of the included studies for this investigation. The study population (POP) consisted of diverse participants, including patients, the general population, healthcare workers, ranchers, and students. The setting of the study was Africa.

### 2.5 Quality assessment

The two authors, YAA and KAG, conducted separate assessments of study quality using the Joanna Briggs Institute (JBI) standardized quality assessment checklist (Aromataris et al., 2017). Our colleague, Natnael Atnafu Gebeyehu, moderated a discussion to resolve discrepancies during the evaluation process. This joint effort ultimately resolved the dispute, resulting in a mutual agreement. The critical analysis checklist consists of eight parameters and offers the selection options Yes, No, Unclear, and Not applicable. Studies were evaluated using quality assessment indicators to determine the risk level. The studies that achieved 50% and above were considered low risk, as indicated in an attached additional file (Supplementary Table S2).

### 2.6 Methodological quality (risk of bias) assessment

We used the methods described by Hoy et al. proposed methods (Hoy et al., 2012) To assess the internal and external validity of the study using 10 specific criteria. These criteria were used to assess the potential risk of bias. Each criterion was assigned either a low or high risk of bias. Articles that lacked precise assessment tools for data collection were classified as high-risk of bias articles. Ultimately, the overall risk of bias was classified as low (total score ≤ 2), moderate (total score between 3 and 4), or high (total score > 5) based on the likelihood of bias (Supplementary Table S3).

### 2.7 Data extraction

YA and KA, two independent authors, used a standardized Microsoft Excel spreadsheet to extract relevant data from the studies. To maintain consistency, they used the JBI prevalence studies tool as a reference point for data extraction from the selected articles (Aromataris et al., 2017). The data extraction tool includes several details, including the author’s name and year of the study, the title of the study, the year of publication, the geographical area and country of the study, the sub-region, the design and type of the study, the study population, the sample size, the response rate, the measured outcome, and the prevalence rate. In addition, information on publication status was also collected.

### 2.8 Data analysis

Data were extracted using Microsoft Excel and analyzed using STATA version 14 statistical software. Significant heterogeneity between studies was assessed using Cochrane Q and I2 statistics. A forest plot was used to represent the heterogeneity visually. A random effects model was used to determine the pooled effect employed as a significant degree of heterogeneity was found. Subgroup analysis was performed based on the subregion, study population, and design. A sensitivity analysis was conducted to examine the influence of a single study on the overall prevalence estimate of the meta-analysis. Publication bias was assessed using funnel plots and Eggers regression tests. If the *p*-value was less than 0.05, it indicated a statistically significant presence of publication bias. The overall effect of knowledge, attitudes, and practices regarding AMR was assessed using a random effects model, and prevalence rates and odds ratios with a 95% confidence interval were calculated.

### 2.9 Search and study selection

**Figure 1:**
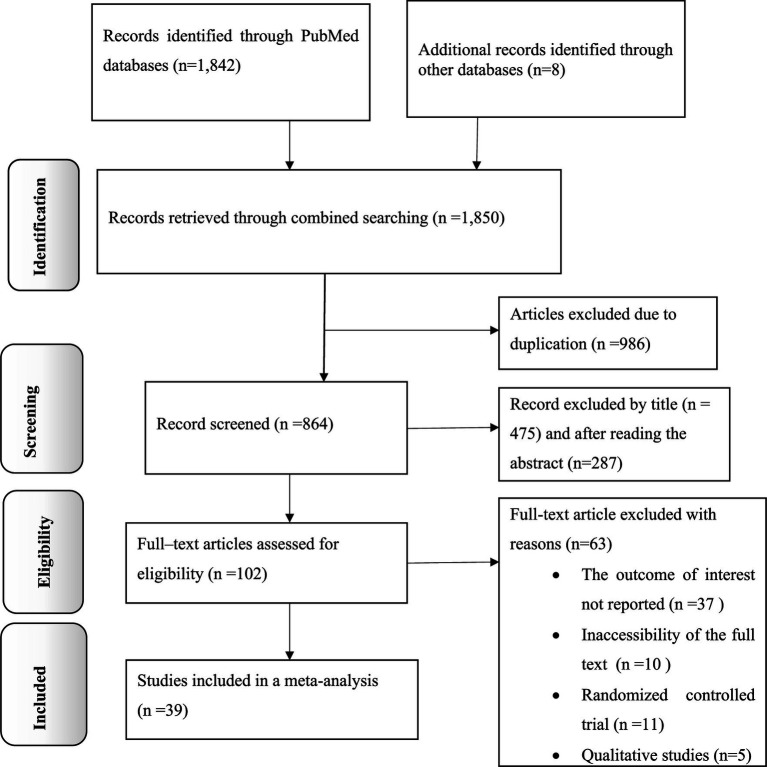
PRISMA flow diagram explaining selection of primary studies in Africa.

The present study is a comprehensive review and meta-analysis focused on examining the dissemination of knowledge, attitudes, and practices related to antimicrobial resistance in Africa. The researchers conducted a thorough search of electronic databases and found a total of 1,850 records. After removing 986 duplicate records, the remaining 864 articles were screened based on their titles and abstracts, excluding 762. Subsequently, 102 full-text articles were carefully assessed for eligibility. Of these, 63 articles were excluded and did not meet the specified criteria. Finally, 39 primary articles were selected for quantitative analysis, representing the most relevant and reliable sources of information for this study (Figure 1).

### 2.10 Study characteristics

This systematic review and meta-analysis included 39 (Thriemer et al., 2013; Abera et al., 2014; Pereko et al., 2015; Simba et al., 2016; Asante et al., 2017; Farley, 2017; Genga et al., 2017; Tafa et al., 2017; El Sherbiny et al., 2018; Seid and Hussein, 2018; Mate et al., 2019; Sunusi et al., 2019; Fetensa et al., 2020; Gemeda et al., 2020; Kabba et al., 2020; Mengesha et al., 2020; Nisabwe et al., 2020; Sindato et al., 2020; Wesangula et al., 2020; Zulu et al., 2020; Aworh et al., 2021; Babatola et al., 2021; Chukwu et al., 2021; Gebeyehu et al., 2021; Geta and Kibret, 2021; Mostafa et al., 2021; Ogoina et al., 2021; Russom et al., 2021; Adegbite et al., 2022; Belachew et al., 2022; Dejene et al., 2022; Kanyike et al., 2022; Mudenda et al., 2022; Paye and McClain, 2022; Tembo et al., 2022; Abubakar and Sárváry, 2023; Davwar et al., 2023; Maarouf et al., 2023; Otieku et al., 2023) published studies involving 18,769 study subjects. The review included primary studies with a remarkably varied sample size, ranging from just 47 (Adegbite et al., 2022) to 2,477 (Russom et al., 2021). Of the included studies, 33 were cross-sectional (Thriemer et al., 2013; Abera et al., 2014; Pereko et al., 2015; Simba et al., 2016; Asante et al., 2017; Farley, 2017; Genga et al., 2017; Tafa et al., 2017; El Sherbiny et al., 2018; Seid and Hussein, 2018; Mate et al., 2019; Fetensa et al., 2020; Gemeda et al., 2020; Mengesha et al., 2020; Nisabwe et al., 2020; Sindato et al., 2020; Zulu et al., 2020; Aworh et al., 2021; Babatola et al., 2021; Chukwu et al., 2021; Gebeyehu et al., 2021; Geta and Kibret, 2021; Mostafa et al., 2021; Ogoina et al., 2021; Russom et al., 2021; Adegbite et al., 2022; Dejene et al., 2022; Kanyike et al., 2022; Mudenda et al., 2022; Paye and McClain, 2022; Abubakar and Sárváry, 2023; Davwar et al., 2023; Maarouf et al., 2023), while six studies followed a cohort study design (Sunusi et al., 2019; Kabba et al., 2020; Wesangula et al., 2020; Belachew et al., 2022; Tembo et al., 2022; Otieku et al., 2023). The study participants for the primary studies were selected by the author from various groups including health workers (Abera et al., 2014; Asante et al., 2017; Genga et al., 2017; Tafa et al., 2017; Kabba et al., 2020; Babatola et al., 2021; Chukwu et al., 2021; Ogoina et al., 2021; Adegbite et al., 2022; Kanyike et al., 2022; Mudenda et al., 2022; Paye and McClain, 2022; Tembo et al., 2022; Abubakar and Sárváry, 2023; Davwar et al., 2023), patients (Farley, 2017; El Sherbiny et al., 2018; Geta and Kibret, 2021; Otieku et al., 2023), Tertiary school students (Thriemer et al., 2013; Seid and Hussein, 2018; Sunusi et al., 2019; Fetensa et al., 2020; Nisabwe et al., 2020; Zulu et al., 2020; Mostafa et al., 2021; Maarouf et al., 2023), community members or the general public (Pereko et al., 2015; Simba et al., 2016; Mate et al., 2019; Mengesha et al., 2020; Sindato et al., 2020; Wesangula et al., 2020; Russom et al., 2021; Belachew et al., 2022; Dejene et al., 2022), and livestock farmers (Farley, 2017; Gebeyehu et al., 2021; Geta and Kibret, 2021; Otieku et al., 2023). Furthermore, The majority of the primary studies included in this review were research reports from East Africa (Abera et al., 2014; Simba et al., 2016; Genga et al., 2017; Tafa et al., 2017; Seid and Hussein, 2018; Fetensa et al., 2020; Gemeda et al., 2020; Mengesha et al., 2020; Nisabwe et al., 2020; Sindato et al., 2020; Wesangula et al., 2020; Zulu et al., 2020; Gebeyehu et al., 2021; Geta and Kibret, 2021; Russom et al., 2021; Belachew et al., 2022; Dejene et al., 2022; Kanyike et al., 2022; Mudenda et al., 2022; Tembo et al., 2022; Table 1).

**Table 1:** The characteristics of the studies included in the systematic review and meta-analysis.

S/N	References	Country	Sub region	Study design	Study population	Sample size	Good knowledge (%)	Positive attitudes (%)	Good practices (%)	QA
1	Abera et al. (2014)	Ethiopia	East Africa	CS	Healthcare workers	385	72.21	NR	NR	9
2	Thriemer et al. (2013)	Congo	Central Africa	CS	Tertiary school students	184	49.50	NR	64.10	9
3	Belachew et al. (2022)	Ethiopia	East Africa	CH	General population	276	76.09	71.74	65.58	8
4	Dejene et al. (2022)	Ethiopia	East Africa	CS	General population	400	35.03	53.75	50.32	9
5	Mostafa et al. (2021)	Egypt	North Africa	CS	Tertiary school students	508	60.51	7.8	39.90	9
6	Gebeyehu et al. (2021)	Ethiopia	East Africa	CS	Livestock producers	571	19.82	14.7	21.51	9
7	El Sherbiny et al. (2018)	Egypt	North Africa	CS	Patients	600	58.03	36.30	53.70	9
8	Gemeda et al. (2020)	Ethiopia	East Africa	CS	Livestock producers	379	18.47	69.39	NR	8
9	Geta and Kibret (2021)	Ethiopia	East Africa	CS	Patients	232	37.51	45.26	43.97	9
10	Mengesha et al. (2020)	Ethiopia	East Africa	CS	General population	374	42.78	50.52	NR	9
11	Maarouf et al. (2023)	Egypt	North Africa	CS	Tertiary school students	626	78	18.21	59	9
12	Seid and Hussein (2018)	Ethiopia	East Africa	CS	Tertiary school students	323	44.89	96.28	NR	9
13	Fetensa et al. (2020)	Ethiopia	East Africa	CS	Tertiary school students	232	68.13	NR	52.01	8
14	Tafa et al. (2017)	Ethiopia	East Africa	CS	Healthcare workers	218	62.84	84.42	NR	9
15	Russom et al. (2021)	Eritrea	East Africa	CS	General population	2,477	39.21	NR	45.00	8
16	Adegbite et al. (2022)	Gabon	Central Africa	CS	Healthcare workers	47	64.00	30	NR	9
17	Otieku et al. (2023)	Ghana	West Africa	CH	Patients	800	NR	NR	39.30	8
18	Asante et al. (2017)	Ghana	West Africa	CS	Healthcare workers	379	80.21	NR	NR	8
19	Paye and McClain (2022)	Liberia	West Africa	CS	Healthcare workers	126	37.90	43.80	64.31	9
20	Wesangula et al. (2020)	Kenya	East Africa	CH	General population	384	75.20	23.02	59.21	9
21	Genga et al. (2017)	Kenya	East Africa	CS	Healthcare workers	107	64.5	NR	36.40	8
22	Mate et al. (2019)	Mozambique	Southern Africa	CS	General population	1,091	46.40	31.00	NR	9
23	Pereko et al. (2015)	Namibia	Southern Africa	CS	General population	446	75.13	42.00	37.00	9
24	Ogoina et al. (2021)	Nigeria	West Africa	CS	Healthcare workers	1,324	22.30	40.30	31.60	9
25	Babatola et al. (2021)	Nigeria	West Africa	CS	Healthcare workers	326	82.74	NR	67.20	8
26	Sunusi et al. (2019)	Sudan	North Africa	CH	Tertiary school students	306	44.44	60.10	NR	9
27	Sindato et al. (2020)	Tanzania	East Africa	CS	General population	816	56.31	41.20	61.50	8
28	Chukwu et al. (2021)	Nigeria	West Africa	CS	Healthcare workers	358	49.20	NR	NR	8
29	Aworh et al. (2021)	Nigeria	West Africa	CS	Livestock producers	144	18.12	25.70	67.40	**9**
30	Simba et al. (2016)	Tanzania	East Africa	CS	General population	1,200	33.70	NR	NR	9
31	Kanyike et al. (2022)	Uganda	East Africa	CS	Healthcare workers	681	87.50	54.40	NR	9
32	Davwar et al. (2023)	Nigeria	West Africa	CS	Healthcare workers	252	41.00	16.00	16.00	9
33	Nisabwe et al. (2020)	Rwanda	East Africa	CS	Tertiary school students	282	96.00	NR	NR	8
34	Farley (2017)	South Africa	Southern Africa	CS	Patients	782	53.00	36.00	45.00	9
35	Zulu et al. (2020)	Zambia	East Africa	CS	Tertiary school students	260	87.30	96.90	75.00	9
36	Tembo et al. (2022)	Zambia	East Africa	CH	Healthcare workers	263	54.40	60.00	64.00	9
37	Mudenda et al. (2022)	Zambia	East Africa	CS	Healthcare workers	178	64.70	81.30	56.60	8
38	Abubakar and Sárváry (2023)	Nigeria	West Africa	CS	Healthcare workers	313	62.30	59.10	64.20	8
39	Kabba et al. (2020)	Sierra Leone	West Africa	CH	Healthcare workers	119	43.70	24.40	NR	9

CS, cross-sectional; CH, cohort; QA, quality assessment; NR, not reported.

### 2.11 Pooled good knowledge regarding antimicrobial resistance in Africa

**Figure 2:**
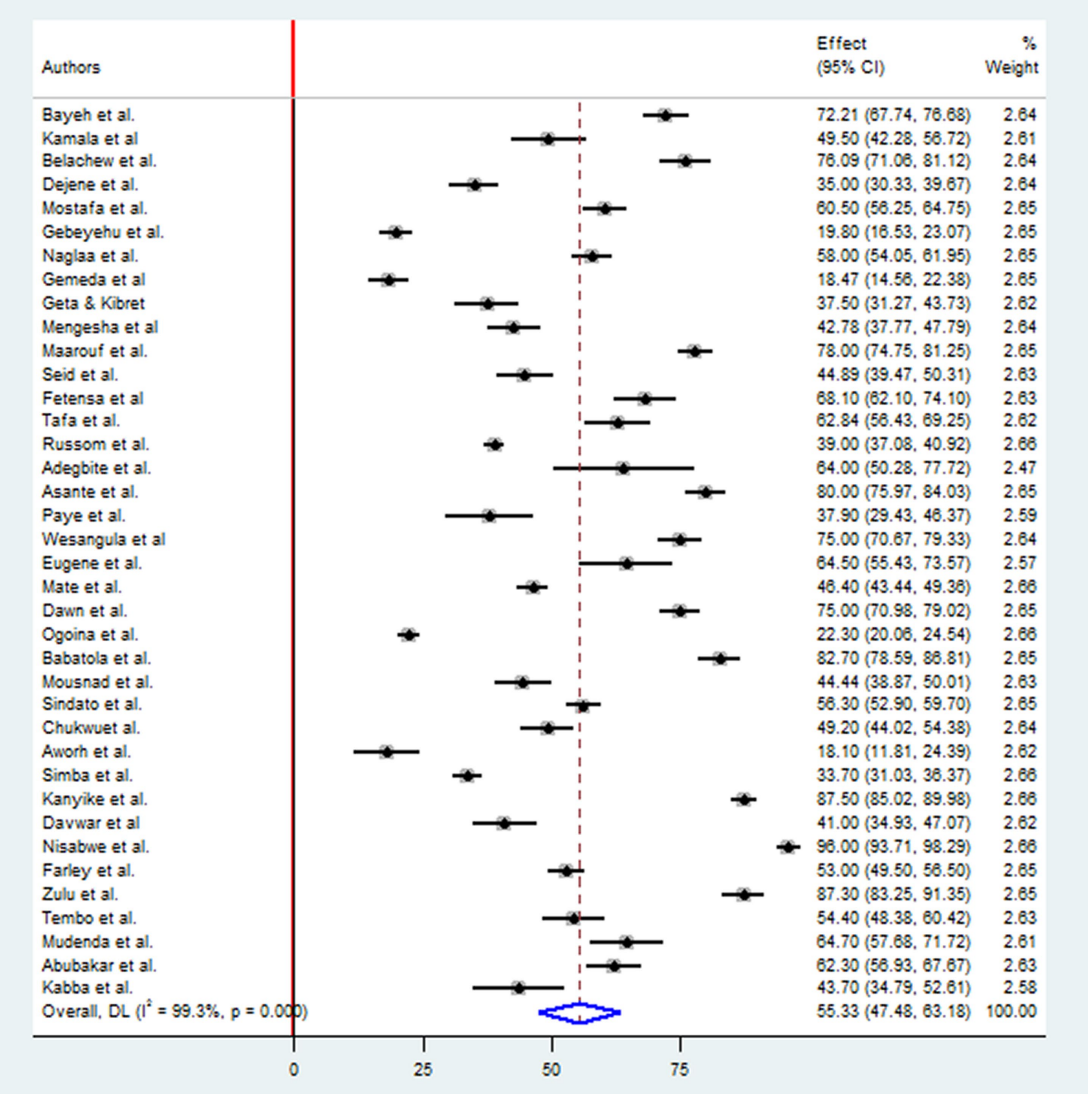
Forest plot for good knowledge toward antimicrobial resistance in Africa.

A comprehensive analysis was conducted on 17,969 participants to determine their knowledge of antimicrobial resistance. The study included 20 research studies in East Africa, nine in West Africa, four in North Africa, three in Southern Africa, and two in Central African countries. The overall pooled prevalence level of knowledge towards antimicrobial resistance in Africa was determined using the random-effect model with Mantel-Hanenszel heterogeneity at 55.33% (95% CI: 47.48, 63.18). This estimate was statistically significant, with a *p*-value of less than 0.001. Furthermore, heterogeneity between studies was found to be high, with an I^2^ value of 99.3% (Figure 2).

### 2.12 Pooled positive attitudes regarding antimicrobial resistance in Africa

**Figure 3:**
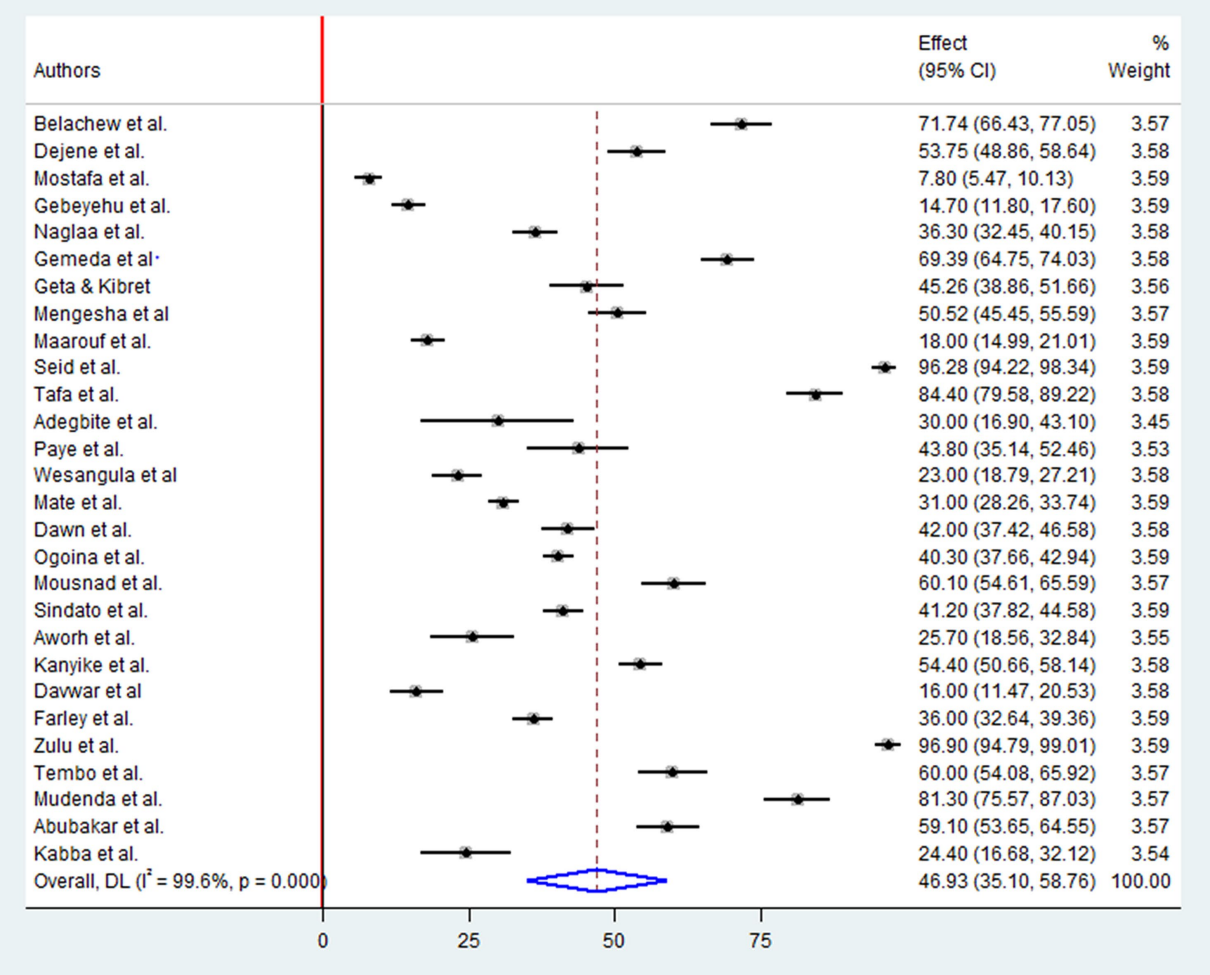
Forest plot for the positive attitude of antimicrobial resistance in Africa.

A Pooled analysis was conducted on a sample of 12,039 participants to determine the level of positive attitudes regarding antibiotic drug resistances. This included 14 studies in East Africa, six in West Africa, four in North Africa, three in Southern Africa, and one in Central Africa. The overall pooled prevalence of positive attitudes toward antimicrobial resistance in Africa was 46.93%(95%CI: 35.10, 58.76), *p* < 0.001, I2 = 99.6% (Figure 3).

### 2.13 Pooled good practices regarding antimicrobial resistance in Africa

**Figure 4:**
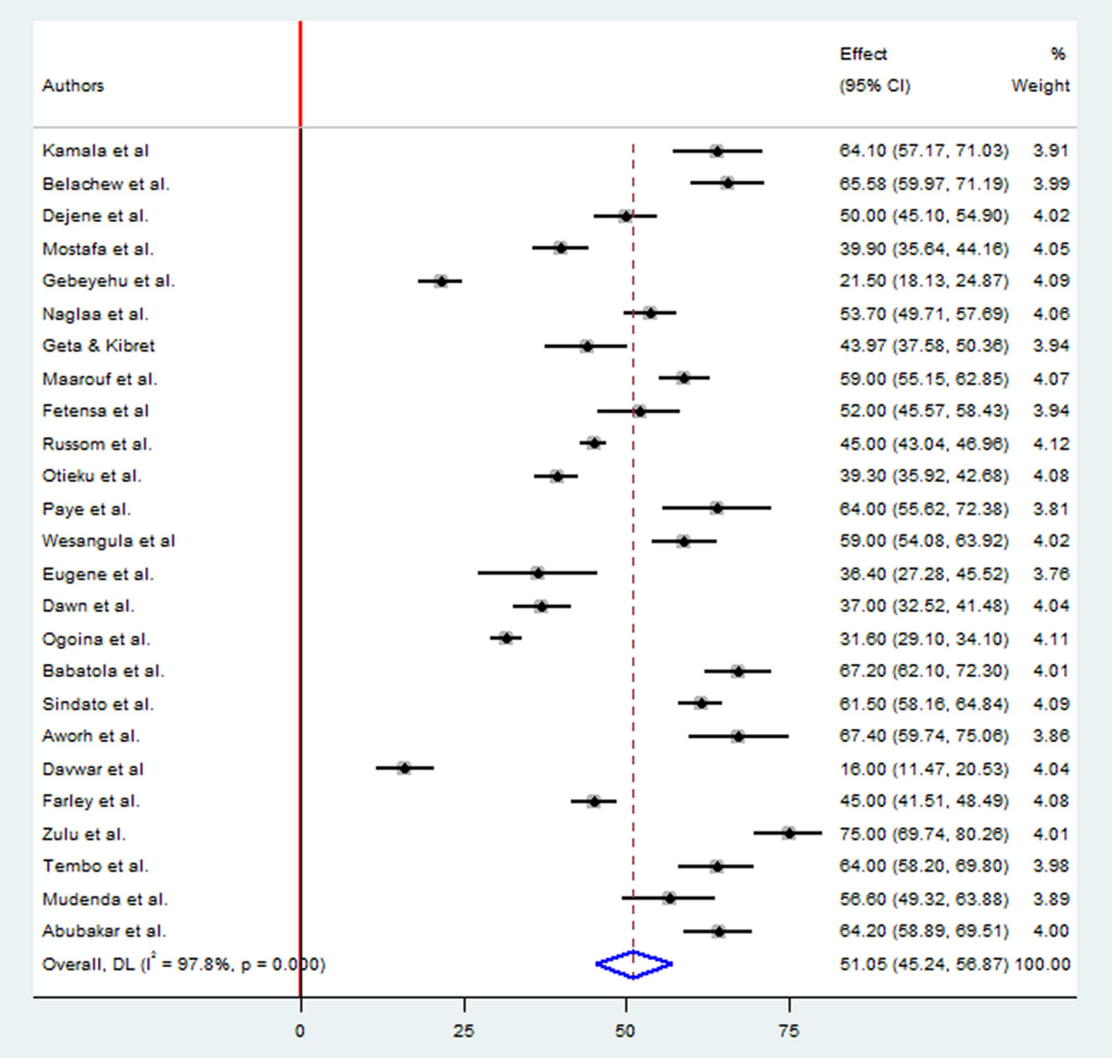
Forest plot for good practice of antimicrobial resistance in Africa.

A total of 12,627 study participants were examined to estimate the level of good practices of antimicrobial drug resistances, which included 12 studies in East Africa, seven studies in West Africa, three studies in Northern Africa, two studies in Southern Africa, and one study in Central Africa country. The overall pooled prevalence of appropriate practice toward antimicrobial resistance in Africa was 51.05% (95%CI: 45.24, 56.87), *p* < 0.001, *I*^2^ = 97.8% (Figure 4).

### 2.14 Sub-group meta-analysis

The subgroup analysis was performed in this meta-analysis by the study population. Among students, it was found that the level of good antimicrobial-resistant knowledge was 66.20% (95% CI: 51.77, 80.64), with the I^2^ statistic from Higgins showing high heterogeneity (I^2^: 99.0%, *p* < 0.001). However, among healthcare workers, the level of good knowledge about antimicrobial resistance was 59.30% (95% CI: 45.19, 73.42), which was also associated with high heterogeneity (I^2^: 99.3%, *p* < 0.001). Likewise, Tertiary school students showed the highest positive attitudes towards antimicrobial resistance, with 55.81% (95% CI: 14.73, 96.90). Higgins and Thompson’s I^2^ statistic showed a significant level of heterogeneity with a *p*-value of less than 0.001. Furthermore, the highest pooled prevalence of good practice was observed among students, with 57.94% (95% CI: 45.78, 70.10). Higgins and Thompson’s I^2^ statistic also showed a significant level of heterogeneity (I^2^:99.5%) with a *p*-value of less than 0.001.

Similarly, Sub-group analysis was conducted to examine the effects of sub-region and study design type. It was found that the study conducted in North Africa had the highest pooled prevalence of good knowledge of antimicrobial resistance with 60.34% (95% CI: 46.76, 73.92); On the other hand, looking at the sub-region, West Africa had the lowest pooled prevalence at 48.62% (95% CI: 30.00, 67.23), and Higgins I^2^ statistic showed a high degree of heterogeneity (I^2^: 97.7%, *p* < 0.001) between studies. Likewise, the highest pooled prevalence of positive attitudes of AMR was observed in the study conducted in East Africa at 60.20% (95% CI: 43.56, 76.85) with high heterogeneity (I^2^:99.6%, *p* < 0.001). Furthermore, a sub-group analysis was based on the study design type. The prevalence among the studies that used cross-sectional study design was relatively higher than those that used cohort study design for each on the level of good knowledge, positive attitudes, and good practices of AMR (Table 2).

**Table 2:** Results of sub-group meta-analysis of good knowledge, positive attitudes, and good practices regards AMR.

Outcomes	Participants characteristics	Included studies	Total participants	Effect size (95% CI)	Heterogeneity	
					I2-value	*P*-value
Good knowledge	Tertiary school students	8	2,721	66.20 (51.77, 80.64)	99.0%	**<**0.001
	Health care workers	15	5,076	59.30 (45.19, 73.42)	99.3%	**<**0.001
	General population	9	7,464	53.20 (42.72, 63.67)	98.8%	**<**0.001
	Patients	3	1,094	49.82 (40.04, 59.60)	93.3%	**<**0.001
	Livestock producers	3	1,614	19.09 (16.76, 21.42)	00.0%	0.830
Total		38		55.33 (47.48, 63.18)	99.3%	<0.001
Good practices	Tertiary school students	5	1810	57.94 (45.78, 70.10)	96.5%	**<**0.001
	General population	6	4,799	52.93 (44.60, 61.25)	96.6%	**<**0.001
	Healthcare workers	8	2,892	49.94 (35.33, 64.56)	98.5%	**<**0.001
	Patients	4	2,414	45.49 (39.12, 51.87)	89.7%	**<**0.001
	Livestock producers	2	715	44.31(−0.66, 89.29)	99.1%	**<**0.001
Total		25		51.05 (45.24, 56.87)	97.8%	<0.001
Positive attitudes	Tertiary school students	5	1,112	55.81 (14.73, 96.90)	99.9%	**<**0.001
	Healthcare workers	10	5,457	49.54 (35.92, 63.16)	98.6%	**<**0.001
	General population	7	2,765	44.64 (33.93, 55.36)	97.9%	**<**0.001
	Patients	3	1,614	38.43 (33.81, 43.05)	70.4%	0.034
	Livestock producers	3	1,094	36.59 (−0.22, 73.41)	99.5%	**<**0.001
Total		28		46.93 (35.10, 58.76)	99.6%	<0.001

### 2.15 Sensitivity meta-analyses

A leave-one-out sensitivity analysis was performed to examine the possible influence of individual studies on the overall pooled prevalence effect. The results showed that no specific study significantly impacted the overall prevalence of good knowledge, positive attitudes, and good antimicrobial resistance (AMR) practices in Africa. The results showed that the combined effect remained significantly unchanged after excluding a specific study.

### 2.16 Meta-regression

A meta-regression analysis was performed to check the presence of statistically significant heterogeneity, as indicated by an I-square test statistic of less than 0.05. The primary objective of this analysis was to determine the underlying source of heterogeneity to ensure an accurate interpretation of the research findings. However, the results of the meta-regression analysis revealed no significant variables that could account for the observed heterogeneity. Specifically, no statistically substantial study-level covariates, such as sample size or publication year, could explain the variations in good knowledge levels, positive attitudes, and good practices towards AMR. Consequently, the heterogeneity observed in this review is likely attributable to other factors not considered in the analysis (Table 3).

**Table 3:** A meta-regression analysis was conducted to examine the factors contributing to the heterogeneity observed between studies.

Heterogeneity sources	Coefficient	Standard error	*P*-value
Level of good AMR knowledge			
Publication year	−0.0130042	0.103559	0.901
Sample size	−0.0002539	0.0004243	0.553
Level of positive attitude toward AMR			
Publication year	−0.1039918	0.1503811	0.495
Sample size	−0.0005252	0.0008723	0.552
Good level of AMR practices			
Publication year	−0.0177752	0.1395804	0.900
Sample size	−0.0000913	0.0004699	0.848

### 2.17 Publication bias (reporting bias)

**Figure 5:**
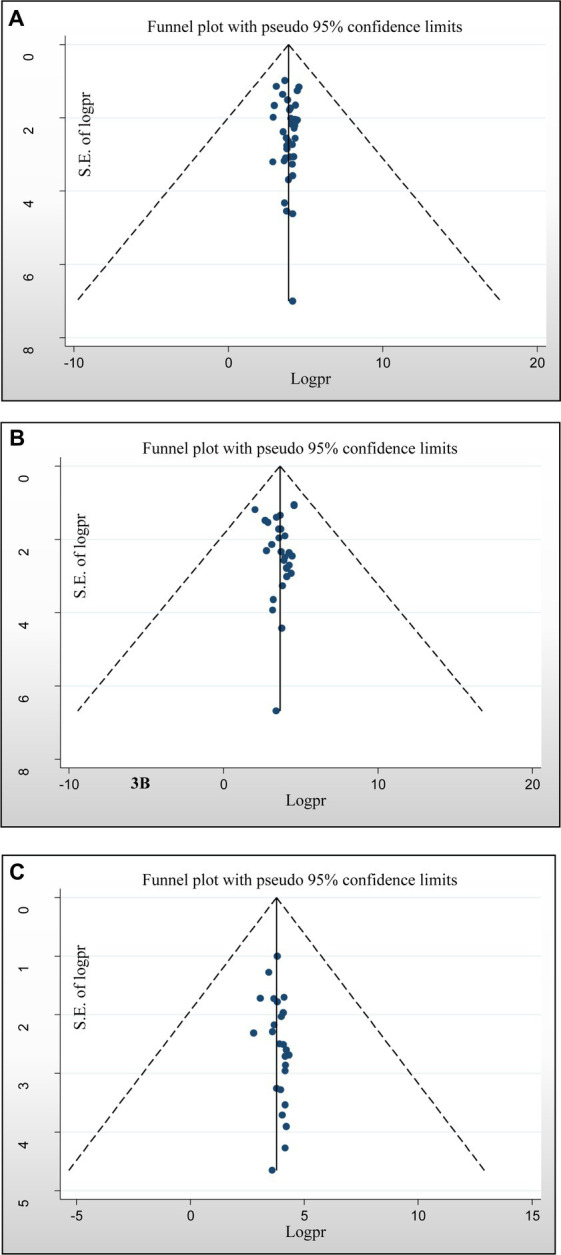
Funnel plots of publication bias. **(A)** For a good level of antimicrobial-resistant knowledge, **(B)** attitudes toward antimicrobial resistance, and **(C)** practices towards antimicrobial resistance.

Assessment of publication bias was performed using subjective and objective approaches. Visual assessment included analysis of a funnel plot, which revealed a slightly asymmetrical distribution of studies. In addition, objective evaluation was performed using Egger’s and Begg’s tests, with the significance level set at *p* < 0.05. The results of the formal Egger linear regression test showed that there was no statistically significant good antimicrobial-resistant knowledge level (*p* = 0.596), positive attitude of antimicrobial resistance (*p* = 0.90), and good practices of AMR (*p* = 0.109). In addition, the presence of publication bias in the included studies was assessed using the Begg regression test. Nevertheless, the results of the Begg regression test showed no evidence of publication bias for the variables good knowledge about antimicrobial resistance (*p* = 0.669), positive attitude towards antimicrobial resistance (*p* = 0.161), and practices (*p* = 0.168) (Figures 5A–C), respectively.

## 3 Discussion

This systematic review and meta-analysis assessed the overall good knowledge, positive attitude, and appropriate practice regarding antimicrobial drug resistance in Africa. Researchers have documented that the prevalence of irrational antimicrobial prescribing and dispensing practices in Africa is often linked to a lack of understanding about antimicrobial resistance (Kamuhabwa and Silumbe, 2013). According to the results, good knowledge was 55.33%, which was in line with the results of the studies on the general rural population in low-income and middle-income Southeast Asia and the Western region of Saudi Arabia (Gualano et al., 2015; Alnasser et al., 2021). However, this finding is significantly lower than research on Japan and Salvan (Kamata et al., 2018; Haenssgen et al., 2019). These different results could be attributed to the commitment to implementing antimicrobial resistance measures, economic status of countries, and their access to antimicrobial resistance information.

This systematic review and meta-analysis found that almost half of participants, particularly over 46.93%, showed a positive attitude towards antimicrobial resistance (AMR). This discovery is consistent with research conducted in southern India (Afzal Khan et al., 2013). This association may be attributed to the fact that more students are aware of antimicrobial drug resistance than the general population, patients, and pet owners, leading to a positive attitude. In addition, we conducted an analysis using data collected from 25 studies involving 12,627 participants. Our findings revealed that the combined proportion of African individuals who adhere to good antimicrobial resistance (AMR) practices was only half the average, at 51.05%.

Furthermore, this proportion was even lower among patients, at 44%. The study identified several prominent poor practices, including dispensing antibiotics without a prescription upon direct request from a client, purchasing antibiotics without consulting a doctor, treating animals with antimicrobials intended for human use, and failing to complete an entire course of antibiotics. These findings align with a review conducted by Gualano MR et al. in Southeast Asia, highlighting the common practices of non-prescribed dispensing of antibiotics in community drug outlets. As reported in this review, the observed low levels of good AMR practices can be attributed to a significant knowledge gap about AMR.

Variations in the level of knowledge regarding antimicrobial drug resistance were observed in different sub-regions of Africa, the highest in Northern Africa (60.34%) and lowest in Western Africa (48.62%). These differences can be attributed to sociocultural, environmental, and economic factors contributing to inequalities in access to already limited knowledge and practices. These factors play a significant role in shaping the observed differences (Gualano et al., 2015).

Furthermore, differences in knowledge related to antimicrobial resistance have been observed in different African study populations. Among these populations, healthcare workers had the highest knowledge about antimicrobial resistance, with 66.20%. On the other hand, animal owners had the lowest level of knowledge at 19.09%. About the general population, this review’s finding was lower than a research report from Thailand and Japan, where approximately half of the public needed more adequate knowledge and awareness about antimicrobial drug resistance (Gualano et al., 2015; Kamata et al., 2018). However, our study’s collective random effect meta-analysis exhibited a higher value than the findings reported in the rural population of low-income and middle-income regions in Southeast Asia and Bhutan (Gualano et al., 2015; Tenzin et al., 2023). The variations in these disparities can be elucidated by considering the number of individuals involved in the study and the execution of the antibiotic stewardship program, along with other associated policies concerning antibiotic utilization and resistance.

In this review, African population groups have different attitudes towards antimicrobial resistance. Among these populations, students showed the highest prevalence of positive attitudes toward antimicrobial resistance, with 55.81%. On the other hand, animal owners showed the lowest pooled prevalence of positive attitudes toward antimicrobial resistance at 36.5%. Notably, these results are lower than those of a study conducted in China, where the prevalence of positive attitudes toward antimicrobial resistance was reported to be 62% (Dyar et al., 2020).

## 4 Limitations of the study

The main limitation of our systematic review and meta-analysis was that the majority of the studies included in this meta-analysis were conducted in East African countries, which may limit the generalizability of our findings to the entire African continent. Furthermore, the determinant factors of the meta-analysis were not pooled due to limited studies that investigated factors associated with knowledge, attitudes, and practices of AMR.

## 5 Conclusion

Findings from this systematic review and meta-analysis revealed a low level of knowledge, attitude, and practice regarding antimicrobial drug resistance among diverse groups, including the general population, patients, students, healthcare workers, and pet owners in Africa. As a consequence, targeted educational interventions need to be in place to raise individuals’ understanding of antimicrobial drug resistance and to develop effective AMR countermeasures.

## Author contributions

YAA: Conceptualization, Investigation, Methodology, Resources, Software, Visualizations, Writing – original draft, Writing – review & editing. KAG: Investigation, Methodology, Supervision, Writing – review & editing, Conceptualization, Data curation.
