# Supplementary material for: Prevalence of knowledge, attitudes, and practices regarding antimicrobial resistance in Africa: a systematic review and meta-analysis
Source: Front Microbiol. 2024 Mar 22;15:1345145. doi: 10.3389/fmicb.2024.1345145 (PMC10996921; doi:10.3389/fmicb.2024.1345145)
Supplement: Supplementary file 2 [file Table_2.docx]

**Supplementary 2**. Quality assessment for the included Studies

| **Item** | **Clearly defined inclusion** | **Describe the study setting and participant** | **Valid and reliable exposure measurement** | **Objective and standard criteria for measurement** | **Identified confounder** | **Strategies to deal with confounders** | **Valid and reliable outcome measurement** | **Appropriate static analysis** | **No of ‘yes’ ‘** |
| --- | --- | --- | --- | --- | --- | --- | --- | --- | --- |
| Nguendo Y. et .al | Yes | Yes | No | Yes | Yes | No | Yes | Yes | 6/8=75 |
| Johnson M.et al | Yes | Yes | Yes | Yes | No | No | Yes | Yes | 6/8=75 |
| Fresenbet F. et al | Yes | Yes | No | Yes | Yes | No | Yes | Yes | 6/8=75 |
| Silamlak B. et .al | Yes | Yes | No | Yes | Yes | Yes | Yes | Yes | 7/8=87.5 |
| Oladoyinbo CA. et | Yes | Yes | No | Yes | Yes | Yes | Yes | Yes | 7/8=87.5 |
| Adhena A. et. al | Yes | Yes | Yes | Yes | Yes | No | Yes | Yes | 7/8=87.5 |
| Belay N. et .al | Yes | Yes | No | Yes | Yes | Yes | Yes | Yes | 7/8=87.5 |
| Sophia S. et .al | Yes | Yes | Yes | Yes | No | No | Yes | Yes | 6/8=75 |
| Garedew T. et .al | Yes | Yes | No | Yes | Yes | Yes | Yes | No | 6/8=75 |
| Lawrence S. et .al | Yes | Yes | Yes | Yes | No | Yes | Yes | No | 6/8=75 |
| Earl SM. et .al | Yes | Yes | Yes | Yes | No | No | Yes | Yes | 6/8=75 |
| Samuel Chane T. | Yes | yes | No | Yes | Yes | Yes | Yes | Yes | 7/8=87.5 |
| Khomotso J. et .al | Yes | Yes | Yes | No | Yes | No | Yes | Yes | 6/8=75 |
| James W. et al | Yes | Yes | Yes | Yes | Yes | Yes | Yes | No | 7/8=87.5 |
| Jember A. et .al | Yes | Yes | Yes | Yes | Yes | Yes | No | Yes | 7/8=87.5 |
| Ndoli D. et .al | Yes | Yes | No | Yes | Yes | Yes | Yes | Yes | 7/8=87.5 |
| Mekuriaw A. et .al | Yes | Yes | No | Yes | No | Yes | Yes | Yes | 6/8=75 |
| Hezron E. et al | Yes | Yes | Yes | Yes | Yes | Yes | Yes | No | 7 /8=87.5 |
| Tegegne A. et .al | Yes | Yes | No | Yes | Yes | No | Yes | Yes | 6/8=75 |
| Fortune A. et .al | Yes | Yes | Yes | Yes | Yes | Yes | Yes | No | 7/8=87.5 |
| Tadege A. et .al | Yes | Yes | Yes | No | Yes | Yes | Yes | No | 6 / 8=75 |
| Ituma B. et .al | Yes | Yes | No | Yes | Yes | Yes | Yes | Yes | 7/8=87.5 |
| Tadesse W. et .al | Yes | Yes | No | Yes | Yes | Yes | Yes | Yes | 7/8=87.5 |
| Odipe OE. et .al | Yes | Yes | No | Yes | Yes | Yes | Yes | Yes | 7/8=87.5 |
| Selepe M. et .al | Yes | Yes | Yes | Yes | Yes | Yes | Yes | No | 7/ 8=87.5 |
| Samuel C. et .al | Yes | Yes | Yes | No | Yes | Yes | No | Yes | 6/8=75 |
| Metadel A. et .al | Yes | Yes | Yes | No | Yes | Yes | Yes | Yes | 7/8=87.5 |
| Penelope T. et .al | Yes | Yes | No | Yes | Yes | Yes | Yes | No | 6/8=75 |
| Henok D. et .al | Yes | Yes | Yes | Yes | Yes | Yes | Yes | No | 7/8=75.5 |
| Abdalla MA. et .al | Yes | Yes | No | Yes | Yes | Yes | No | Yes | 7/8=75.5 |
| Jane Sebolelo N. et al | Yes | Yes | Yes | Yes | Yes | No | Yes | Yes | 7/8=75.5 |
| Lesiba A. et .al | Yes | Yes | Yes | Yes | No | Yes | Yes | No | 6/8=75 |
| Kate B. et .al | Yes | Yes | No | Yes | Yes | Yes | No | Yes | 6/8=75 |
| Agerie M. et .al | Yes | Yes | Yes | Yes | Yes | Yes | No | Yes | 7/8=75.5 |
| Tessema A. et .al | Yes | Yes | No | Yes | Yes | Yes | Yes | Yes | 7/8=75.5 |
| Sanbato T. et .al | Yes | Yes | No | Yes | Yes | No | Yes | Yes | 6/8=75 |
| Mariam O. et .al | Yes | Yes | No | Yes | Yes | No | Yes | Yes | 6/8=75 |
| Dawit G. et .al | Yes | Yes | Yes | Yes | Yes | Yes | No | Yes | 7/8=75.5 |
| Limbikani M. et .al | Yes | Yes | Yes | Yes | Yes | Yes | No | Yes | 7/8=75.5 |
| Okojie OH. et .al | Yes | Yes | Yes | Yes | No | Yes | No | Yes | 6/8=75 |
| Isara A. et .al | Yes | Yes | Yes | No | Yes | Yes | Yes | Yes | 7/8=75.5 |
| Omemu M. et .al | Yes | Yes | No | Yes | Yes | No | Yes | Yes | 6/8=75 |
