# Supplementary material for: Prevalence of knowledge, attitudes, and practices regarding antimicrobial resistance in Africa: a systematic review and meta-analysis
Source: Front Microbiol. 2024 Mar 22;15:1345145. doi: 10.3389/fmicb.2024.1345145 (PMC10996921; doi:10.3389/fmicb.2024.1345145)
Supplement: Supplementary file 3 [file Table_3.docx]

**Supplementary 3**. Risk of bias assessment for the included studies

| **Item** | **External validity** | | | | **Internal validity** | | | | | |  | |
| --- | --- | --- | --- | --- | --- | --- | --- | --- | --- | --- | --- | --- |
|  | Representativeness of the target population | Representativeness of the sampling frame | Radom sampling or census | Minimal response bias | Data were collected directly | Acceptable case definition used in the study | Valid and reliable measurement t | The same mode of data collection for all study subject | Appropriate length of prevalence period for parameter of interest | Appropriate numerators and denominator s of interest | No of yes | Sum ery of risk of bias |
| Nguendo Y. et .al | Yes | Yes | Yes | Yes | No | No | Yes | Yes | Yes | Yes | 8 | Low- risk |
| Johnson M.et al | Yes | Yes | No | Yes | Yes | No | Yes | Yes | Yes | Yes | 8 | Low- risk |
| Fresenbet F. et al | Yes | Yes | No | Yes | Yes | Yes | Yes | Yes | Yes | Yes | 9 | Low – risk |
| Silamlak B. et .al | Yes | Yes | No | Yes | Yes | Yes | Yes | Yes | Yes | Yes | 9 | Low- risk |
| Oladoyinbo CA. et | Yes | Yes | Yes | Yes | Yes | No | Yes | Yes | Yes | Yes | 9 | Low- risk |
| Adhena A. et. al | Yes | Yes | No | Yes | Yes | Yes | Yes | Yes | Yes | Yes | 9 | Low- risk |
| Belay N. et .al | Yes | Yes | Yes | Yes | No | No | Yes | Yes | Yes | Yes | 8 | Low- risk |
| Sophia S. et .al | Yes | Yes | No | Yes | Yes | Yes | Yes | No | Yes | Yes | 8 | Low- risk |

| Garedew T. et .al | Yes | Yes | Yes | Yes | No | Yes | Yes | No | Yes | Yes | 8 | Low- risk |
| --- | --- | --- | --- | --- | --- | --- | --- | --- | --- | --- | --- | --- |
| Lawrence S. et .al | Yes | Yes | Yes | Yes | No | No | Yes | Yes | Yes | Yes | 8 | Low-risk |
| Earl SM. et .al | Yes | yes | No | Yes | Yes | Yes | Yes | Yes | Yes | Yes | 9 | Low- risk |
| Samuel Chane T. | Yes | Yes | Yes | No | Yes | No | Yes | Yes | Yes | Yes | 8 | Low-risk |
| Khomotso J. et .al | Yes | Yes | Yes | Yes | Yes | Yes | Yes | No | Yes | Yes | 9 | Low-risk |
| James W. et al | Yes | Yes | Yes | Yes | Yes | Yes | No | Yes | Yes | Yes | 8 | Low- risk |
| Jember A. et .al | Yes | Yes | No | Yes | Yes | Yes | Yes | Yes | Yes | Yes | 9 | Low risk |
| Ndoli D. et .al | Yes | Yes | Yes | Yes | Yes | Yes | Yes | Yes | No | No | 8 | Low- risk |
| Mekuriaw A. et .al | Yes | Yes | Yes | Yes | Yes | Yes | Yes | No | No | Yes | 8 | Low- risk |
| Hezron E. et al | Yes | Yes | Yes | Yes | Yes | Yes | Yes | Yes | Yes | No | 9 | Low- risk |
| Tegegne A. et .al | Yes | Yes | Yes | Yes | Yes | Yes | Yes | No | No | Yes | 8 | Low -risk |
| Fortune A. et .al | Yes | Yes | Yes | No | Yes | Yes | Yes | No | Yes | Yes | 8 | Low -risk |
| Tadege A. et .al | Yes | Yes | Yes | No | Yes | Yes | Yes | Yes | Yes | Yes | 9 | Low-risk |
| Ituma B. et .al | Yes | Yes | Yes | Yes | Yes | Yes | Yes | No | Yes | No | 8 | Low-risk |
| Tadesse W. et .al | Yes | Yes | Yes | No | No | Yes | Yes | Yes | Yes | Yes | 8 | Low-risk |
| Odipe OE. et .al | Yes | Yes | Yes | Yes | No | Yes | Yes | Yes | Yes | Yes | 9 | Low-risk |
| Selepe M. et .al | Yes | No | Yes | No | Yes | Yes | Yes | Yes | Yes | Yes | 8 | Low-risk |
| Samuel C. et .al | Yes | Yes | Yes | Yes | Yes | Yes | Yes | No | Yes | Yes | 9 | Low-risk |
| Metadel A. et .al | Yes | Yes | Yes | Yes | No | Yes | Yes | Yes | No | Yes | 8 | Low-risk |
| Penelope T. et .al | Yes | Yes | No | Yes | Yes | Yes | Yes | Yes | Yes | Yes | 8 | Low-risk |
| Henok D. et .al | Yes | Yes | Yes | Yes | Yes | No | Yes | Yes | Yes | Yes | 9 | Low-risk |
| Abdalla MA. et .al | Yes | Yes | Yes | Yes | Yes | Yes | No | Yes | Yes | Yes | 9 | Low-risk |
| Jane Sebolelo N. et al | Yes | Yes | Yes | Yes | Yes | Yes | Yes | No | Yes | No | 8 | Low-risk |
| Lesiba A. et .al | Yes | No | Yes | Yes | No | Yes | Yes | Yes | Yes | Yes | 9 | Low-risk |
| Kate B. et .al | Yes | Yes | Yes | Yes | Yes | Yes | Yes | Yes | No | Yes | 9 | Low-risk |
| Agerie M. et .al | Yes | Yes | Yes | No | Yes | No | Yes | Yes | Yes | Yes | 8 | Low-risk |
| Tessema A. et .al | Yes | Yes | Yes | Yes | Yes | Yes | No | Yes | Yes | Yes | 9 | Low-risk |
| Sanbato T. et .al | Yes | Yes | No | Yes | Yes | Yes | Yes | No | Yes | Yes | 8 | Low-risk |
| Mariam O. et .al | Yes | Yes | Yes | No | Yes | Yes | Yes | Yes | Yes | Yes | 9 | Low-risk |
| Dawit G. et .al | Yes | Yes | Yes | Yes | Yes | Yes | Yes | No | Yes | Yes | 9 | Low-risk |
| Limbikani M. et .al | Yes | Yes | Yes | Yes | No | Yes | Yes | Yes | No | Yes | 8 | Low-risk |
| Okojie OH. et .al | Yes | Yes | Yes | Yes | Yes | No | Yes | Yes | Yes | Yes | 9 | Low-risk |
| Isara A. et .al | Yes | Yes | Yes | Yes | Yes | Yes | Yes | No | Yes | Yes | 9 | Low-risk |
| Omemu M. et .al | Yes | Yes | Yes | Yes | No | Yes | Yes | Yes | No | Yes | 8 | Low-risk |
